# Standardization of SARS-CoV-2 Cycle Threshold Values: Multisite Investigation Evaluating Viral Quantitation across Multiple Commercial COVID-19 Detection Platforms

**DOI:** 10.1128/spectrum.04470-22

**Published:** 2023-01-18

**Authors:** Kenneth Gavina, Lauren C. Franco, Christopher M. Robinson, Weston Hymas, Guang-Sheng Lei, Will Sinclair, Tara Hall, John Carlquist, John-Paul Lavik, Christopher L. Emery, Phillip R. Heaton, David Hillyard, Bert K. Lopransi, Ryan F. Relich

**Affiliations:** a Department of Pathology and Laboratory Medicine, Indiana University School of Medicine, Indianapolis, Indiana, USA; b Division of Clinical Microbiology, Indiana University Health, Indianapolis, Indiana, USA; c Department of Microbiology and Immunology, Indiana University School of Medicine, Indianapolis, Indiana, USA; d ARUP Institute for Clinical and Experimental Pathology, Salt Lake City, Utah, USA; e Intermountain Laboratory Services, Department of Pathology, Intermountain Healthcare, Salt Lake City, Utah, USA; f Department of Pathology and Laboratory Medicine, Health Partners, Bloomington, Minnesota, USA; Johns Hopkins Hospital

**Keywords:** SARS-CoV-2, harmonization, standardization, quantitation, viral load

## Abstract

The demand for testing during the coronavirus disease 2019 (COVID-19) pandemic has resulted in the production of several different commercial platforms and laboratory-developed assays for the detection of severe acute respiratory syndrome coronavirus 2 (SARS-CoV-2). This has created several challenges, including, but not limited to, the standardization of diagnostic testing, utilization of cycle threshold (*C_T_*) values for quantitation and clinical interpretation, and data harmonization. Using reference standards consisting of a linear range of SARS-CoV-2 concentrations quantitated by viral culture-based methods and droplet digital PCR, we investigated the commutability and standardization of SARS-CoV-2 quantitation across different laboratories in the United States. We assessed SARS-CoV-2 *C_T_* values generated on multiple reverse transcription-PCR (RT-PCR) platforms and analyzed PCR efficiencies, linearity, gene targets, and *C_T_* value agreement. Our results demonstrate the inappropriateness of using SARS-CoV-2 *C_T_* values without established standards for viral quantitation. Further, we emphasize the importance of using reference standards and controls validated to independent assays, to compare results across different testing platforms and move toward better harmonization of COVID-19 quantitative test results.

**IMPORTANCE** From the onset of the COVID-19 pandemic, the demand for SARS-CoV-2 testing has resulted in an explosion of analytical tests with very different approaches and designs. The variability in testing modalities, compounded by the lack of available commercial reference materials for standardization early in the pandemic, has led to several challenges regarding data harmonization for viral quantitation. In this study, we assessed multiple commercially available RT-PCR platforms across different laboratories within the United States using standardized reference materials characterized by viral culture methods and droplet digital PCR. We observed variability in the results generated by different instruments and laboratories, further emphasizing the importance of utilizing validated reference standards for quantitation, to better harmonize SARS-CoV-2 test results.

## INTRODUCTION

Reverse transcription-PCR (RT-PCR) has become the laboratory gold standard for the diagnosis of coronavirus disease 2019 (COVID-19). Multiple PCR platforms and assays have been developed to detect severe acute respiratory syndrome coronavirus 2 (SARS-CoV-2) RNA in clinical specimens using different gene targets, chemistries, and extraction methods. Although it has been approved for the qualitative detection of SARS-CoV-2, some organizations report the cycle threshold (*C_T_*) value as a correlative quantitative measure of viral load, with the rationale that this information could be beneficial in predicting disease progression, prognosis, and viral infectivity (1–4). Other organizations, including the College of American Pathologists (CAP), have cautioned against the reporting of *C_T_* values due to the nonstandardization of test methods and variabilities in sample collection, sample transportation, RNA extraction, gene targets, and PCR efficiency, all of which ultimately influence *C_T_* values (5).

Studies have compared *C_T_* values for identical samples among different assays and laboratories. As part of an external quality assessment, Buchta et al. compared *C_T_* values generated from 5 identical clinical samples that were tested on 101 distinct RT-PCR platforms (6). They reported slight intraprotocol variations in *C_T_* values and more substantial interprotocol variations (36.5% of tests deviated from the mean by ±2.0 cycles or more, and 7.7% of tests deviated from the mean by ±4.0 cycles or more). The authors recommended limitations on the interpretation of *C_T_* values and highlighted the need to standardize *C_T_* values by gene target, at the very least (6). Similarly, the CAP used proficiency testing data to compare *C_T_* values reported for identical sample material from >700 laboratories and found that the median *C_T_* values reported by different assays varied by as much as 14.0 cycles (5).

In this study, we assessed SARS-CoV-2 *C_T_* values generated across multiple sites and platforms to investigate the standardization and commutability of SARS-CoV-2 quantitation. This work was performed prior to the establishment of the World Health Organization (WHO) international standard for SARS-CoV-2 RNA (National Institute for Biological Standards and Control [NIBSC] code 20/146). Instead, control material consisted of a linear range of SARS-CoV-2 quantities, which were quantitated by viral culture-based methods and droplet digital PCR (ddPCR). We highlight the importance of appropriate reference standards and specific considerations for the harmonization of SARS-CoV-2 quantitation in the context of the comparison of laboratory results and for informed decision-making in clinical settings.

## RESULTS

### Quantitation of viral culture.

Serial dilutions, ranging from 1e5 to 1e−1 PFU/mL and from 1.8e5 to 1e−1 50% tissue culture infective dose (TCID_50_)/mL, were prepared by plaque assay and endpoint dilution, respectively, and were used for absolute quantitation by ddPCR and assessment of *C_T_* value variations across different RT-quantitative PCR (qPCR) platforms (Table 1). Due to the dynamic range limitations of ddPCR, only serial dilutions of 1e3 to 1e0 PFU/mL (1.8e3 to 1.83e0 TCID_50_/mL) were tested. Viral copies for serial dilutions outside this range were calculated by using the slope from a generated line of best fit for nucleocapsid 1 (N1) and N2. Viral copies of the dilution series ranged from 9.6e4 to 1.6e−1 copies/μL and from 9.1e4 to 1.7e−1 copies/μL for N1 and N2, respectively, when quantitated by ddPCR. The linearities for the N1 and N2 genes were identical (*R*^2^ = 0.999).

**TABLE 1:** Summary of viral standard quantitation by different methods

Dilution concentration		ddPCR result (viral copies/μL) for^*a*^:		RT-qPCR *C_T_*^*b*^	
PFU/mL	TCID_50_/mL				
		N1	N2	Median (IQR)	Range
1e5	1.8e5	9.6e4	9.1e4	14.8 (12.5–15.6)	11.7–16.8
1e4	1.8e4	1.0e4	1.0e4	18.6 (16.1–18.9)	15.4–20.2
1e3	1.8e3	1.0e3	1.0e3	21.3 (19.4–22.2)	18.9–23.0
1e2	1.8e2	1.2e2	1.2e2	24.5 (22.7–25.4)	22.3–26.2
1e1	1.8e1	2.1e1	2.2e1	27.6 (25.8–28.6)	24.9–29.3
1e0	1.8e0	1.1e0	1.2e0	29.9 (29.2–31.6)	28.7–32.1
1e−1	1.8e−1	1.6e−1	1.7e−1	33.0 (32.3–34.3)	31.1–37.1

a Copies per microliter for 1e5, 1e4, and 1e−1 standards were calculated by using the slope from the line of the best fit for N1 and N2.

b Medians, IQRs, and ranges were determined using *C_T_* values from replicates on all platforms from all participating sites.

### Evaluation of individual RT-qPCR assays.

**FIG 1:**
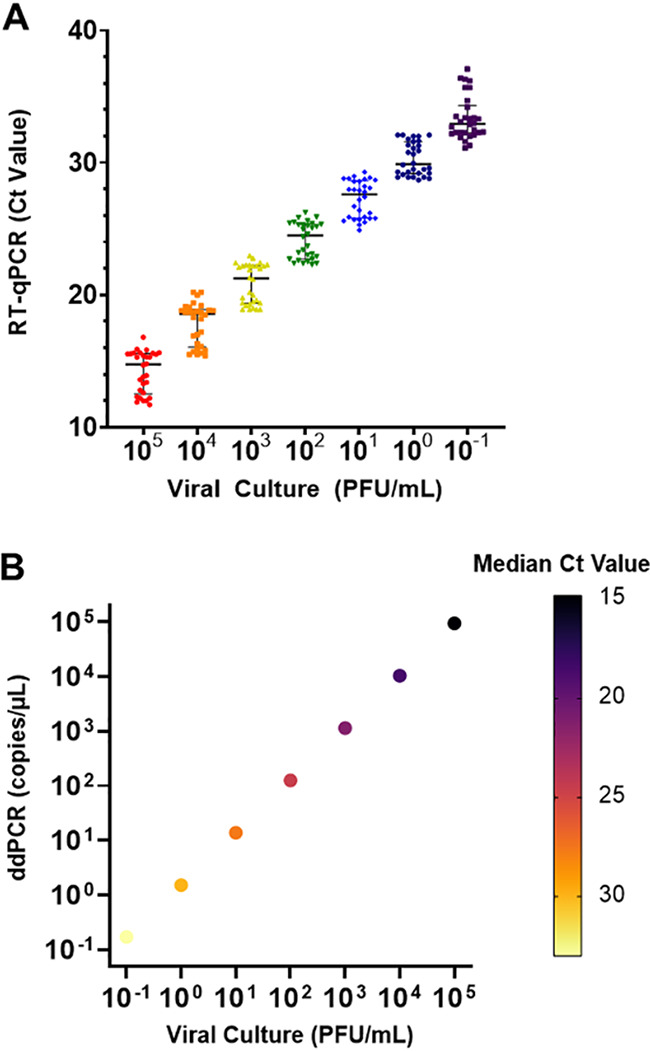
Quantitation of SARS-CoV-2 viral loads by RT-qPCR. (A) Variation of SARS-CoV-2 *C_T_* values assessed on different RT-qPCR platforms across different institutions. (B) SARS-CoV-2 *C_T_* values assessed by two independent quantitative measures, i.e., ddPCR and viral culture.

The full range, median, and interquartile range (IQR) of *C_T_* values from all platforms at each testing site are shown in Table 1. The differences in median *C_T_* values between dilutions averaged 3.0 cycles, and the median IQRs did not overlap the immediately higher or lower dilution. However, the full range of *C_T_* values for each dilution overlapped with the adjacent dilutions by an average of 0.7 cycles, with the largest overlap being 1.3 cycles and the smallest being 0.6 cycles. The distribution of *C_T_* values across all sites and platforms was plotted against viral culture serial dilutions (Fig. 1A). Within a single titer, *C_T_* values among all instruments and sites differed by up to 6.0 cycles. The minimum range of *C_T_* values reported for a single titer was 3.4 cycles and, on average, *C_T_* values within a single titer differed by an average of 4.5 cycles. The relationships among viral culture titers, quantitation by ddPCR, and median *C_T_* values are shown in Fig. 1B. Quantitation by ddPCR was consistent with culture-based titers; increasing titers and the numbers of gene copies per microliter both correlated with decreasing *C_T_* values, demonstrating overall linearity across the different quantitative methods.

### Evaluation of *C_T_* values by different RT-qPCR platforms at multiple sites.

We compared the sample volumes, gene targets, PCR efficiency, and *R*^2^ values among the different SARS-CoV-2 platforms and sites (Table 2). Input sample volumes for the different platforms ranged from 50 μL (DiaSorin) to 750 μL (BD MAX). Gene targets in the platforms compared in this study included the N1, N2, envelope (E), spike (S), open reading frame 1 (ORF1), and RNA-dependent RNA polymerase (RdRP) genes. PCR efficiency ranged from 96.8% to 136.7%. The Xpert Xpress CoV-2/Flu/respiratory syncytial virus (RSV) *plus* assay was the only assay run at all three sites, and it had efficiencies of 103.7%, 97.75, and 96.8% at sites 1, 2, and 3, respectively. The cobas SARS-CoV-2 test, which was run at sites 2 and 3, had high efficiencies for both of its targets (ORF1 and E) at both sites (136.7% for ORF1 and 117.6% for E at site 2 and 113.3% for ORF1 and 123.4% for E at site 3). The efficiencies of the remaining platforms ranged from 97.8% to 109.5%, with the Thermo Fisher Scientific TaqMan SARS-CoV-2 assay performing closest to 100% for all three targets (ORF1, 100.4%; N, 102.9%; S, 100.0%). The Panther Fusion SARS-CoV-2 assay was also among those closest to 100% efficiency for its single target, ORF1. The ORF1 target is a single target for four of the platforms (DiaSorin, Panther Fusion, cobas, and Thermo Fisher Scientific) included in this study and was included in assays run by all three sites. Efficiencies for this target ranged from 100.4% to 136.7%. All assays had *R*^2^ values of ≥0.99, with the exception of the cobas SARS-CoV-2 test ORF1 target assay that was run at site 2 (*R*^2^ = 0.98).

**TABLE 2:** Summary of PCR performance characteristics for different SARS-CoV-2 detection platforms across different testing sites

Site and platform	Sample volume (μL)	Target	Efficiency (%)	*R* ^2^
Site 1				
BD Max	750	N1	97.8	0.999
BD Max	750	N2	106	0.999
Cepheid	300	E, N2, RdRP	103.7	0.998
DiaSorin	50	S	108.9	0.990
DiaSorin	50	ORF1	109.5	0.990
Panther Fusion	500	ORF1	102.6	0.999
Site 2				
Cepheid	300	E, N2, RdRP	97.7	0.997
cobas	650	ORF1	136.7	0.980
cobas	650	E	117.6	0.991
Site 3				
Cepheid	300	E, N2	96.8	0.999
cobas	650	ORF1	113.3	0.995
cobas	650	E	123.4	0.993
Thermo Fisher Scientific	200	ORF1	100.4	0.999
Thermo Fisher Scientific	200	N	102.9	0.999
Thermo Fisher Scientific	200	S	100	0.999

Assays performed at each of the sites were directly compared to one another, and the *C_T_* value mean bias for every comparison is shown in Table 3. Thirty-nine percent of the comparisons had a mean bias of <1.00, 24.8% had a mean bias between 1.00 and 1.99, 26.8% had a mean bias between 2.00 and 2.99, and 9.5% had a mean bias of >2.99. The BD MAX N1 assay at site 1, the Xpert Xpress CoV-2/Flu/RSV *plus* assay at site 2, and the Thermo Fisher Scientific TaqMan SARS-CoV-2 ORF1 and S assays at site 3 had *C_T_* mean bias values of >1.99 for >50% of the assays to which they were compared. The Xpert Xpress CoV-2/Flu/RSV *plus* assay was run at all three sites and had *C_T_* mean bias values of 1.06 (site 1 versus site 2), 0.84 (site 1 versus site 3), and 0.21 (site 2 versus site 3) when the three sites were compared to one another.

**TABLE 3:**
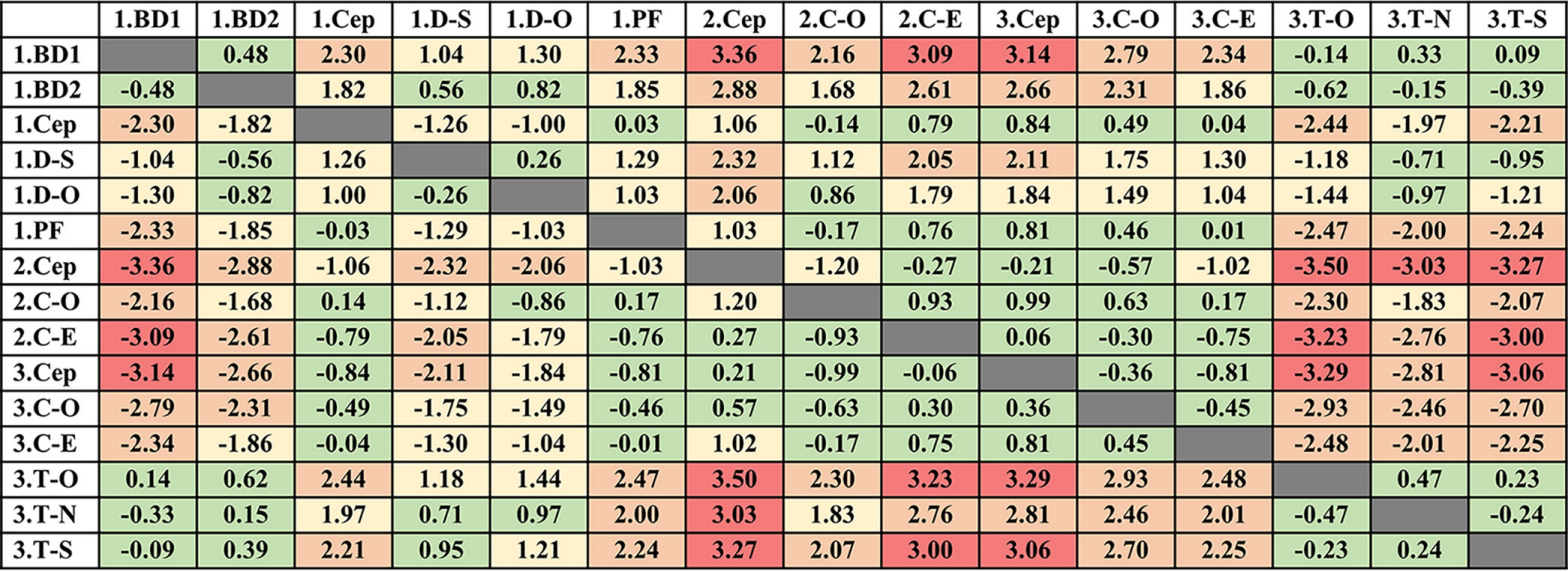
Summary of the Bland-Altman analysis assessing *C_T_* agreement across different sites and SARS-CoV-2 detection platforms^*a*^

a Green, mean bias of <1.00; yellow, mean bias of 1.00 to 1.99; orange, mean bias of 2.00 to 2.99; red, mean bias of ≥3.00; 1.BD1, site 1, BD MAX N1 target; 1.BD2, site 1, BD MAX N2 target; 1.Cep, site 1, Cepheid; 1.D-S, site 1, DiaSorin S target; 1.D-O, site 1, DiaSorin ORF1 target; 1.PF, site 1, Panther Fusion; 2.Cep, site 2, Cepheid; 2.C-O, site 2, cobas ORF1 target; 2.C-E, site 2, cobas E target; 3.Cep, site 3, Cepheid; 3.C-O, site 3, cobas ORF1 target; 3.C-E, site 3, cobas E target; 3.T-O, site 3, Thermo Fisher Scientific ORF1 target; 3.T-N, site 3, Thermo Fisher Scientific N target; 3.T-S, site 3, Thermo Fisher Scientific S target.

## DISCUSSION

Since the inception of the COVID-19 pandemic, the high demand for SARS-CoV-2 testing has resulted in an explosion of analytical tests with highly different approaches and designs (i.e., commercial versus in-house, automated versus manual, and singleplex versus multiplex). The variability in available testing methods, compounded by the lack of standardization or available commercial reference material early in the pandemic, is enough to suggest a lack of homogeneity in the *C_T_* value results that are reported by most clinical laboratories (7).

Many studies have evaluated the appropriateness of SARS-CoV-2 *C_T_* values for qualitative testing versus quantitative testing (7–14); despite this, however, several studies have also been published in which health care providers encourage the reporting of *C_T_* values for the purposes of patient care decision-making and public health policy implementation (e.g., isolation, discharge, and quarantine protocols) (1–4, 15). In a study by Magleby et al., 678 hospitalized patients with COVID-19 were retrospectively assessed with SARS-CoV-2 *C_T_* values, and results were correlated with clinical outcomes such as severe disease and death (3). Other published studies have used *C_T_* values to directly correlate outcomes and associations such as dysosmia/dysgeusia, headache, immunoglobulin G status, risk of intubation, hospital admission mortality rates, cancer-associated mortality rates, and viral load dynamics (13, 16–19). Clinical laboratories report *C_T_* values to requesting physicians to help guide the course of clinical action. However, regarding the assessment of transmission and infection control, studies investigating the correlation between *C_T_* values and infectious SARS-CoV-2 have shown conflicting results (20–23), suggesting the inappropriateness of using *C_T_* values to extrapolate and establish a cutoff value for virus infectivity and clinical significance.

Prior to the availability of the WHO SARS-CoV-2 international standard, there have been a few studies that have investigated the commutability of testing and variability of *C_T_* values. Using data collected from CAP proficiency tests from 700 different laboratories across the United States, Rhoads et al. demonstrated variance in *C_T_* values of up to 12 to 14 cycles among different U.S. Food and Drug Administration (FDA)-approved emergency use authorization (EUA) methods (14). Another study found high interprotocol variability of various SARS-CoV-2 assays by evaluating three semiquantitated positive samples (high [average *C_T_* value of 36.3 cycles], medium [average *C_T_* value of 28.9 cycles], and low [average *C_T_* value of 24.8 cycles]) and comparing the *C_T_* values based on the various gene targets (6). A study conducted by Vierbaum et al. utilized two different reference materials (10^7^ copies/mL and 10^6^ copies/mL) quantitated by ddPCR to assess *C_T_* value variance across >300 different laboratories in Germany (7).

In our study, we evaluated SARS-CoV-2 viral load quantitation and *C_T_* value variability across different instrument platforms and institutions, using a linear range of control material that was quantitated by two viral infectivity assays and ddPCR. The use of a dilution series, as opposed to a single concentration or semiquantitated concentrations, allowed us to assess linearity and PCR efficiency for the different platforms and gene targets, as well as to compare *C_T_* values through a full range of viral quantities. When the data from all sites and platforms were analyzed, *C_T_* values differed with a range of 3.4 to 6.0 cycles for the different SARS-CoV-2 dilutions tested. Individual assay comparisons of *C_T_* value mean biases across all testing platforms and sites showed no trends or associations with the SARS-CoV-2 gene target, PCR efficiency, or input sample volume. This finding suggests that, despite the standardization of sample input for each assay, it is highly recommended that each site perform its own analysis of correlations between generated *C_T_* values for each instrument and a quantitated standard to compare results across different targets, platforms, and sites. It has been well established that SARS-CoV-2 *C_T_* values may differ based on the gene target due to various factors, including PCR efficiency based on amplicon product length, differences in stringency in the binding of the various target-specific primers, and gene expression and/or availability in the specimen being tested (7, 14, 24). Importantly, these findings may also be highly transferrable to other quantitative viral assays commonly used in the clinical microbiology laboratory (e.g., cytomegalovirus [CMV], herpes simplex virus [HSV], and Epstein-Barr virus [EBV]).

Our study is not without limitations; our control material was generated from a single SARS-CoV-2 USA-WA1/2020 isolate, and our assessment does not take into consideration sequence differences observed in the different SARS-CoV-2 variants (e.g., variants of concern [VOCs]). While a WHO international standard for SARS-CoV-2 RNA (NIBSC code 20/146) is now commercially available (25), use of this material would involve similar challenges, compared to our current control material, in that this reference standard would not take into account future VOCs or changes within the genome sequence. For test commutability and harmonization of data, it would be prudent to have an adjustable, or modular, international standard that may be modified to incorporate relevant sequence changes in emerging VOCs. Further, it is reasonable to presume that the findings from our study may in fact underestimate the degree of test commutability, particularly when the multitude of variables associated with real patient specimens is taken into consideration. Another study limitation is that viral genome integrity and the potential for fragmentation from collected clinical specimens were not taken into consideration. It is known that fragmentation of nucleic acid directly impacts the accuracy of molecular quantitation (26–28), a proposed mechanism by which the availability of sequence for a target-specific primer is compromised due to fragmentation of the nucleic acid sample. While we are unable to account for fragmentation within our control material, the issue presents a greater challenge for standardization of SARS-CoV-2 quantitation using a control with equally fragmented control material, which requires further investigation into whether this is a necessity for quantitation and, more importantly, whether it is technically feasible.

Our work demonstrates the quantitative capabilities of different commercial SARS-CoV-2 RT-PCR assays, but with the emphasis that correlation between *C_T_* values and viral loads be made only with established, quantitated control material. Further, we describe the inappropriateness of the commutability of SARS-CoV-2 *C_T_* values across different platforms and sites due to the inherent variability in results. While the utilization of reference standards and control materials is a necessary tool that may allow us to compare results across different tests, there remain many challenges for the harmonization of SARS-CoV-2 viral loads that may not be accounted for only by employing a control reference standard.

## MATERIALS AND METHODS

### Quantitation of SARS-CoV-2 standards by viral infectivity assays.

Quantitative standards were prepared using a stock of passage 6 SARS-CoV-2 isolate USA-WA1/2020 (BEI Resources, Manassas, VA), propagated in Cercopithecus aethiops kidney cells (Vero E6; ATCC, Manassas, VA). The titer of the stock was determined by plaque assay and endpoint dilution assay using Vero E6 cells according to standard methods, and titers were determined to be 1.95 × 10^6^ (1.95e6) PFU/mL and 3.58 × 10^6^ (3.58e6) TCID_50_/mL, respectively (29, 30). Stocks were then aliquoted, heat inactivated at 65°C for 30 min, removed from high containment, and frozen at −80°C until use. All work was performed in a biosafety level 3 laboratory following institutional biosafety committee-approved and validated protocols from the Indiana University School of Medicine.

### Preparation of quantitative standards.

Quantitative standards were prepared from the viral culture stocks described above. The culture was serially diluted 10-fold in a naso-oropharyngeal viral transport medium matrix. The matrix consisted of pooled naso-oropharyngeal patient specimens from previously tested samples that were SARS-CoV-2 negative by the Roche cobas SARS-CoV-2 assay (cobas 8800; Roche, Indianapolis, IN). A total of seven serial dilutions were prepared, covering the range of 1e5 to 1e−1 PFU/mL (1.84e5 to 1.84e−1 TCID_50_/mL). Serial dilution aliquots were frozen at −80°C until further downstream analysis.

### Quantitation by ddPCR.

Viral nucleic acid was extracted from 200 μL of heat-inactivated SARS-CoV-2 stock using the chemagic MSM I system (PerkinElmer, Santa Clara, CA) and eluted in a total volume of 80 μL. ddPCR was performed using the one-step RT-ddPCR advanced kit for probes (Bio-Rad, Hercules, CA) with the 2019-nCoV CDC ddPCR triplex probe assay (catalog number dEXS28563542; Bio-Rad) targeting the N1 and N2 genes, following the manufacturer’s protocol. Briefly, droplet generation was performed with the AutoDG system (Bio-Rad), and amplification was performed with a C1000 thermocycler (Bio-Rad); plates were read with a QX200 droplet reader (Bio-Rad), and results were analyzed manually using QuantaSoft Analysis Pro v1.0.596 (Bio-Rad).

### Quantitation by RT-qPCR.

The detection of SARS-CoV-2 by RT-qPCR was performed using multiple platforms across three different sites to assess *C_T_* variability among different institutions and instruments. All standards were tested in duplicate; screening assays were performed according to the manufacturers’ protocols, with default software baseline subtraction and *C_T_* value determination. At site 1 (HealthPartners, Bloomington, MN), RT-qPCR was performed using the Xpert Xpress CoV-2/Flu/RSV *plus* kit (Cepheid, Sunnyvale, CA), the Simplexa COVID-19 direct kit (performed using the Liaison MDX thermocycler; DiaSorin, Cypress, CA), the SARS-CoV-2 assay for the Panther Fusion system (Hologic, Santa Clara, CA), and the SARS-CoV-2 assay for the BD MAX system (Becton, Dickinson and Company, Franklin Lakes, NJ). At site 2 (Indiana University Health, Indianapolis, IN), RT-qPCR was performed using the Xpert Xpress CoV-2/Flu/RSV *plus* assay and the cobas SARS-CoV-2 test (performed using the cobas 8800 system; Roche). At site 3 (Intermountain Medical Center; Murray, UT), RT-qPCR was performed using the Xpert Xpress CoV-2/Flu/RSV *plus* assay, the cobas SARS-CoV-2 test (performed using the cobas 6800 system), and the TaqMan SARS-CoV-2 assay (QuantStudio; Thermo Fisher Scientific, Carlsbad, CA).

### Statistical analysis.

Statistical analyses and graphical presentations were generated using Prism v9 (GraphPad, San Diego, CA). PCR efficiencies and coefficients of correlation (*R*^2^) were calculated from the quantitative standards for each individual platform by plotting *C_T_* values against serial dilutions and performing a Deming regression analysis. For comparisons of *C_T_* values across different gene targets, a D’Agostino-Pearson omnibus normality test was used to determine whether data followed a normal distribution. To investigate the agreement between different platforms and sites, we performed a Bland-Altman analysis and plotted average quantitation against the difference in quantitation for the serial dilutions tested.
